# Moderating effect of negative emotion differentiation in chronic stress and fatigue among Chinese employees

**DOI:** 10.3389/fpsyg.2024.1358097

**Published:** 2024-05-23

**Authors:** Wenhao Lv, Huake Qiu, Hongliang Lu, Zhang Yajuan, Ma Yongjie, Chen Xing, Xia Zhu

**Affiliations:** Department of Military Medical Psychology, Air Force Medical University, Xi'an, China

**Keywords:** stress, fatigue, anxiety, depression, emotion differentiation

## Abstract

**Introduction:**

According to the reactivity hypothesis and the diathesis-stress model, repeated activation of the stress system has a negative effect on health, and this effect may differ because of individual characteristics. Thus, the present study explores the effect of chronic stress on fatigue and investigates its mechanism.

**Methods:**

A questionnaire survey of 288 participants selected from the northwest part of China was conducted (13.89% females; ages ranged from 18 to 34 years, with *M* ± *SD* = 23.14 ± 3.79 years) on chronic stress, fatigue, depression, anxiety, and negative emotion differentiation. SPSS 28.0 was used to process descriptive statistics and correlation analysis and the PROCESS macro was used to analyze the moderated chained multi-mediation.

**Results:**

Chronic stress was found to be positively correlated with fatigue, depression, and anxiety; depression and anxiety played a chained multi-mediating role between chronic stress and fatigue, and negative emotion differentiation played a moderating role in the chained multi-mediation model.

**Discussion:**

Compared with depression, anxiety plays a more important role in the influence of chronic stress on fatigue. Therefore, it is necessary to pay more attention to anxiety symptoms and take appropriate intervention measures. Negative emotion differentiation plays a moderating role. Improving negative emotion differentiation through mindfulness and adaptive emotion regulation is an effective way to reduce the influence of chronic stress on fatigue.

## 1 Introduction

With the development of the social economy, people feel environmental pressures such as lengthening working hours. This kind of stress is a long-term presence in people’s daily lives, and thus constitutes chronic stress. Stress caused by external stressors consists of non-adaptive physiological, behavioral, and cognitive responses (Koolhaas et al., 2011). Stress can be divided into acute stress and chronic stress, both of which typically have negative effects on health (O’Connor et al., 2021).

Many researchers have studied the effects of acute stress. Physiologically, acute stress can induce changes in the connectivity of large-scale resting state networks (such as default mode networks) and inactivation of the hippocampus, all of which are related to the cortisol response (Zhang et al., 2019; Corr et al., 2021). In addition, acute stress plays an important role in social decision-making. Acute stress promotes prosocial behaviors, especially sharing with others, but does not affect risk behaviors (von Dawans et al., 2019). Clinically, cognitive behavior therapy (CBT) can effectively prevent post-traumatic stress disorder [PTSD (Bryant, 2018)] subsequent to acute stress.

The related studies on chronic stress have mainly focused on the influencing factors of various diseases, such as cardiovascular diseases (Iob and Steptoe, 2019), Alzheimer’s disease (Bisht et al., 2018) and oral diseases (Decker et al., 2021). Moreover, chronic stress affects individuals’ cognition and increase their susceptibility to psychological problems (Marin et al., 2011). Chronic stress has more influence on depression than acute stress, and chronic stress can moderate the relationship between acute stress and depression (McGonagle and Kessler, 1990). This indicates that chronic stress can also significantly affect health.

Studies have demonstrated the influence of chronic stress on fatigue. However, the relationship between chronic stress and fatigue is related to many factors, and there have been few studies on the variables that play moderating roles between chronic stress and fatigue. Therefore, this study deeply explores the psychological mechanism by which chronic stress affects fatigue and explores the moderating role of other factors in the influence of chronic stress.

### 1.1 Effect of chronic stress on fatigue

Several studies have shown that stress can affect fatigue. Higher chronic stress can affect fatigue the next day by reducing sleep quality (Astill et al., 2013; Doerr et al., 2015). Workers with high working stress are more likely to have fatigue (Yogisutanti et al., 2020). Subjective fatigue and stress change together in the daily life of married couples (Doerr et al., 2018). However, the relationship between stress and fatigue has mainly been studied using physiological indexes such as cortisol and α amylase. Studies to date have explored the relationship between stress and fatigue, but have seldom looked into the psychological influence mechanism of that relationship.

According to the reactivity hypothesis, repeated activation of the stress system leads to adverse health outcomes (Krantz and Manuck, 1984). In a long-term stress environment, there are persistent changes in adaptive physiological indexes (such as cortisol), and repeated changes like these put the biological system out of balance. This leads to allostatic overload, fatigue, and adverse health effects (McEwen, 2017). To sum up, this study hypothesizes as follows:

*H1*: Chronic stress affects fatigue and the higher the chronic stress, the higher the fatigue.

### 1.2 Mediating role of depression and anxiety

During the COVID-19 pandemic, chronic stress was related to depression and anxiety (Varma et al., 2021), depression (Mayer et al., 2018) and anxiety (Juruena et al., 2020) increased with the increase in stress. At the same time, in a persistent stress environment, acute life events are closely related to severe depression (McGonagle and Kessler, 1990; Hammen et al., 2009). Chronic stress affects depressive symptoms by affecting sleep quality (Da Estrela et al., 2021). In addition, chronic stress affects fatigue on the following day by affecting sleep quality (Doerr et al., 2015), which indicates that there may be a correlation between depressive symptoms and fatigue. Thus, chronic stress may affect fatigue through depressive symptoms.

A large number of studies have shown that depression and anxiety both have negative effects on fatigue. Among patients with chemotherapy-induced sensory peripheral neuropathy (CIPN), patients with severe anxiety and depression had more fatigue (Bonhof et al., 2019). Women with generalized anxiety disorder (GAD) report greater fatigue than healthy women (Li et al., 2020). Individuals with higher trait anxiety have stronger social media fatigue (SMF) in terms of cognition, behavior, and emotion (Swiatek et al., 2021). Chronic stress is related to anxiety and depression, and anxiety and depression can affect fatigue. Thus, this study hypothesizes as follows:

*H2*: Depression and anxiety play a mediating role between chronic stress and fatigue.

### 1.3 Moderating role of negative emotion differentiation

Li et al. (2020) found that the increase of mental fatigue during the female luteal phase may be negatively affected by impaired emotional regulation. Thus, emotion-related abilities may play an important moderating role in the generation and increase of fatigue. Emotion differentiation refers to the fact that there are differences in the ways individuals experience emotions (Barrett et al., 2001). Individuals with low emotion differentiation cannot easily distinguish the specific emotions they experience, whereas individuals with high emotion differentiation can use specific emotional words to describe their emotions (i.e., identifying an emotion as anger rather than sadness). Emotion differentiation is divided into negative and positive emotion differentiation according to valence. Some studies have paid more attention to the influence of negative emotion differentiation (Lischetzke et al., 2021). Depression and anxiety are negative emotions, and so this study takes into account negative emotion differentiation.

According to the diathesis-stress model, stress is an important factor in promoting the development of various psychological problems, but not all people will have psychological problems in response to a stressful environment. This suggests that traits and abilities play important roles in coping with stress (Monroe and Simons, 1991). The moderating role of negative emotion differentiation between stress and depression has been studied in different populations. Negative emotion differentiation moderates the relationship between stress and depression in veterans, while it does not moderate that relationship in college students (Starr et al., 2017). At the same time, emotion differentiation has been shown to moderate depression and anxiety by promoting adaptive emotional regulation in adolescence (Nook et al., 2021). However, adults also face great stress, but few studies have examined emotion differentiation in the working population. Therefore, it is necessary to study the moderating role of negative emotion differentiation in the negative effects of stress for different subjects. Thus, this study hypothesizes as follows:

*H3*: Negative emotion differentiation affects the relationship between chronic stress and fatigue by moderating the anxiety and depression caused by chronic stress. Compared with those with low negative emotion differentiation, individuals with high negative emotion differentiation are less likely to be affected by the chronic stress that results in anxiety and depression, and then affects fatigue.

**Figure 1:**
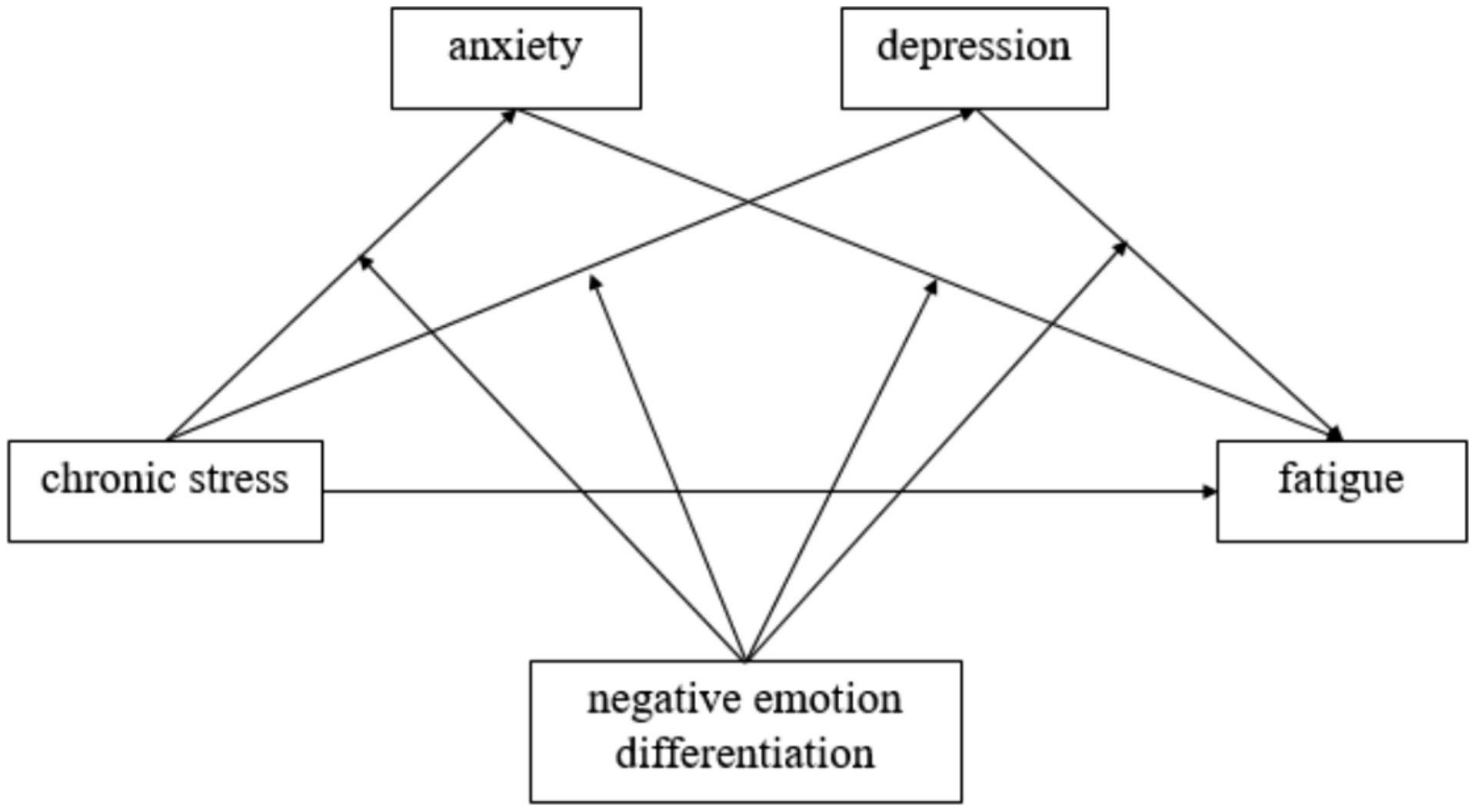
Hypothesized model.

The hypothesized model is shown in Figure 1.

## 2 Methods

### 2.1 Participants

A convenience sampling approach was used in our study to recruit targeted participants and finally 315 employees were randomly selected from the northwest part of China. Excluding 27 participants who chose the same responses on successive multiple scales or had inappropriately short answer times, the remaining participants were 288 in total (13.89% females; ages ranged from 18 to 34 years, with *M* ± *SD* = 23.14 ± 3.79 years). The effective response rate was 91.43%. All respondents were right-handed, with normal intelligence and no dyslexia, volunteered to participate, and signed the informed consent. The data collection procedures were approved by the Ethics Committee of Tangdu Hospital.

### 2.2 Procedure

Questionnaires were distributed to all participants in the same period of time. An online survey was used, with the questionnaire link published through Wechat. In order to ensure the authenticity and accuracy of the data, each participant only answered once through the link. Before the start of the study, the researchers promised the participants that the experimental data of this study would be kept confidential and that all the data would only be used for scientific research.

First, participants completed the questions on demographic information. Next, participants completed the Perceived Stress Scale (PSS), the Epworth Sleeping Scale (ESS), and the Depression Anxiety and Stress Scale (DASS-21). Finally, the participants completed the negative emotion differentiation measurement.

### 2.3 Materials

#### 2.3.1 Negative emotion differentiation

Following the example of previous studies, this study used a standard laboratory-based task to measure negative emotion differentiation (Erbas et al., 2014; Nook et al., 2018, 2021). In this study, participants were presented with 20 negative pictures selected from the Open Affective Standardized Image Set (OASIS) (Kurdi et al., 2017). Each picture was presented for 5 s, and participants rated a battery of emotions (anger, shame, disgust, sadness, and fear) on a 10-point scale (from 1 = *not at all* to 10 = *extremely*) (Nook et al., 2021). There was no time limit for the ratings. Negative emotion differentiation was measured by calculating the average intra-class correlation coefficients of the ratings of the 20 negative pictures. The lower the ICCs, the higher the negative emotion differentiation (Erbas et al., 2014; Widdershoven et al., 2019). In order to express negative emotion differentiation conveniently, the final scores of the ICCs were subtracted from one, to represent the negative emotion differentiation of the participants. The higher the score, the stronger the negative emotion differentiation ability.

#### 2.3.2 Chronic stress

The PSS was used to measure the chronic stress of the participants over the past month (Cohen et al., 1983). This scale consists of 14 items. Participants rated the degree to which each item reflected their experience on a 5 – point scale (from 1 = *not at all* to 5 = *extremely*). Among them, positive scoring was used for items 1, 2, 3, 8, 11, 12, and 14, while reverse scoring was used for items 4, 5, 6, 7, 9, 10, and 13. The higher the score, the more chronic stress was perceived. In the current study, the Cronbach’s α for this scale was 0.73 and the construct validity was 0.88.

#### 2.3.3 Fatigue

Fatigue was assessed with ESS (Johns, 1991). This scale comprises 8 items scored on a 4 – point scale (from 0 = *Never napping or falling asleep* to 5 = *More likely to nap or fall asleep*). All items were positively scored, with a higher score indicating higher fatigue. On the ESS, less than 5 is normal, 5–9 is mild sleepiness, 10–15 is moderate sleepiness, and 16–24 is severe sleepiness. In this study, the Cronbach’s α for the ESS was 0.76 and the construct validity was 0.75.

#### 2.3.4 Depression and anxiety

The subscales of depression and anxiety in the DASS-21 were used to measure the degrees of depression and anxiety of the participants (Lovibond and Lovibond, 1996; Gong et al., 2010). The depression subscale and the anxiety subscale each comprise 7 items, scored on a 4 – point scale (from 0 = *Strongly disagree* to 5 = *Strongly agree*). All items were positively scored, with a higher score indicating a higher level of depression or anxiety. For the depression subscale, the Cronbach’s α was 0.90, and the construct validity was 0.91. For the anxiety subscale, the Cronbach’s α was 0.84, and the construct validity was 0.88.

### 2.4 Data analysis and common method bias test

Descriptive statistics and correlation analyses of all data were processed through SPSS 28.0. The PROCESS macro in SPSS 28.0 was used to analyze chained multi-mediation and moderated chained multi-mediation (Hayes, 2012).

## 3 Results

### 3.1 Common method bias

Harman’s single factor test was used to test for common method bias. The results show that 8 factors were generated without rotation, and the variance interpretation percentage of the first common factor was 30.03% (less than 40%), so there was no obvious common method bias in this study.

### 3.2 Description and correlation analyses

Descriptive statistics and correlation analyses were made on the differentiation of chronic stress, fatigue, depression, anxiety, and negative emotions (Table 1). The results indicate that chronic stress was positively correlated with fatigue, depression, and anxiety. Moreover, chronic stress was moderately correlated with depression and anxiety, fatigue was moderately correlated with depression and anxiety, and the correlation coefficient between depression and anxiety was strong.

**Table 1:** Descriptive statistics and correlations for all variables.

	M	SD	1	2	3	4	5
Chronic stress	21.67	7.43	1				
Fatigue	7.75	4.21	0.25^***^	1			
Depression	2.24	3.59	0.48^***^	0.42^***^	1		
Anxiety	2.78	3.61	0.47^***^	0.45^***^	0.81^***^	1	
**Negative** emotion differentiation	0.69	0.30	−0.06	−0.04	−0.02	−0.02	1

M = mean. SD = standard deviations. ^*^p < 0.05; ^**^ *p* < 0.01; ^***^ *p* < 0.001.

### 3.3 The mediating role of depression and anxiety

To further reveal the psychological mechanism by which chronic stress affects fatigue, chronic stress was taken as the independent variable, depression and anxiety as mediating variables, and fatigue as the dependent variable. The PROCESS macro (Model 6) in SPSS 28.0 was used to analyze the chain multiple mediating effects (Hayes, 2012). The results in Table 2 indicate that chronic stress could positively predict fatigue (*β* = 0.25, *t* = 4.35, *p* < 0.001), anxiety (*β* = 0.48, *t* = 9.26, *p* < 0.001), and depression (*β* = 0.11, *t* = 2.76, *p* < 0.01). Anxiety could significantly positively predict depression (*β* = 0.75, *t* = 19.29, *p* < 0.001). After adding the mediating variables, the positive predictive effect of chronic stress on fatigue was not significant (*β* = 0.03, *t* = 0.48, *p* = 0.63), while that of anxiety was significant (*β* = 0.31, *t* = 3.41, *p* < 0.001), and that of depression was critical (*β* = 0.15, *t* = 1.68, *p* = 0.095).

**Table 2:** Mediation analysis.

Regression equation		Overall fitting index			Regression coefficient	
Outcome variable	Predictive variable	*R*	*R* ^2^	*F*(df)	*β*	*t*
Anxiety		0.48	0.23	85.70_(1)_^***^		
	Chronic stress				0.48	9.26^***^
Depression		0.81	0.66	279.84_(2)_^***^		
	Chronic stress				0.11	2.76^**^
	Anxiety				0.75	19.29^***^
Fatigue		0.25	0.06	18.89_(1)_^***^		
	Chronic stress				0.25	4.35^***^
Fatigue		0.46	0.21	25.13_(3)_^***^		
	Chronic stress				0.03	0.48
	Anxiety				0.31	3.41^***^
	Depression				0.15	1.68

All variables in the model were entered into the regression equation after standardization. ^**^ *p* < 0.01; ^***^ *p* < 0.001.

Further mediating effect analysis (see Table 3) showed that the indirect effect of depression and anxiety was significant (*β* = 0.22, SE = 0.04, 95% CI = 0.13 to 0.30), accounting for 88% of the total effect. Anxiety had a significant mediating effect between chronic stress and fatigue (*β* = 0.03, SE = 0.02, 95% CIs = [0.01, 0.07]), and depression and anxiety had a significant chain mediating effect between chronic stress and fatigue (*β* = 0.11, SE = 0.04, 95% CIs = [0.04, 0.19]).

**Table 3:** Testing the pathways of the mediation model.

	*β*	SE	95% confidence interval	
			Lower	Upper
Total effect	0.25	0.06	0.00	0.13
Direct effect	0.03	0.06	−0.09	0.15
Indirect effect	0.22	0.04	0.13	0.30
Ind 1	0.07	0.04	−0.01	0.16
Ind 2	0.03	0.02	0.01	0.07
Ind 3	0.11	0.04	0.04	0.19

Ind 1: chronic stress – > depression – > fatigue; Ind 2: chronic stress – > anxiety – > fatigue; Ind 3: chronic stress – > anxiety – > depression – > fatigue.

### 3.4 The moderating role of negative emotion differentiation

To assess the moderating effect of negative emotion differentiation, chronic stress was taken as the independent variable, depression and anxiety as mediating variables, fatigue as the dependent variable, and negative emotion differentiation as the modulating variable. The PROCESS macro (Model 91) in SPSS 28.0 was used to reveal the moderating role of negative emotion differentiation in the relationship between anxiety and depression, and to construct a moderated chain mediation model (Hayes, 2012). Regression analysis (see Table 4) showed that the interaction between anxiety and negative emotion differentiation had a significant predictive effect on depression (*β* = −0.08, *t* = −2.49, *p* < 0.05).

**Table 4:** Moderated mediation analysis.

Regression equation		Overall fitting index			Regression coefficient	
Outcome variable	Predictive variable	*R*	*R* ^2^	*F*(df)	*β*	*t*
Anxiety		0.47	0.22	81.72_(1)_^***^		
	Chronic stress				0.47	9.04^***^
Fatigue		0.46	0.21	25.13_(3)_^***^		
	Chronic stress				0.03	0.48
	Anxiety				0.31	3.41^***^
	Depression				0.15	1.68
Depression		0.82	0.67	145.86_(4)_^***^		
	Chronic stress				0.14	3.52^***^
	Anxiety				0.73	18.36^***^
	Negative emotional differentiation				0.01	0.29
	Anxiety*negative emotional differentiation				−0.08	−2.49^*^

All variables in the model were entered into the regression equation after standardization. ^*^ *p* < 0.05; ^***^ *p* < 0.001.

**Figure 2:**
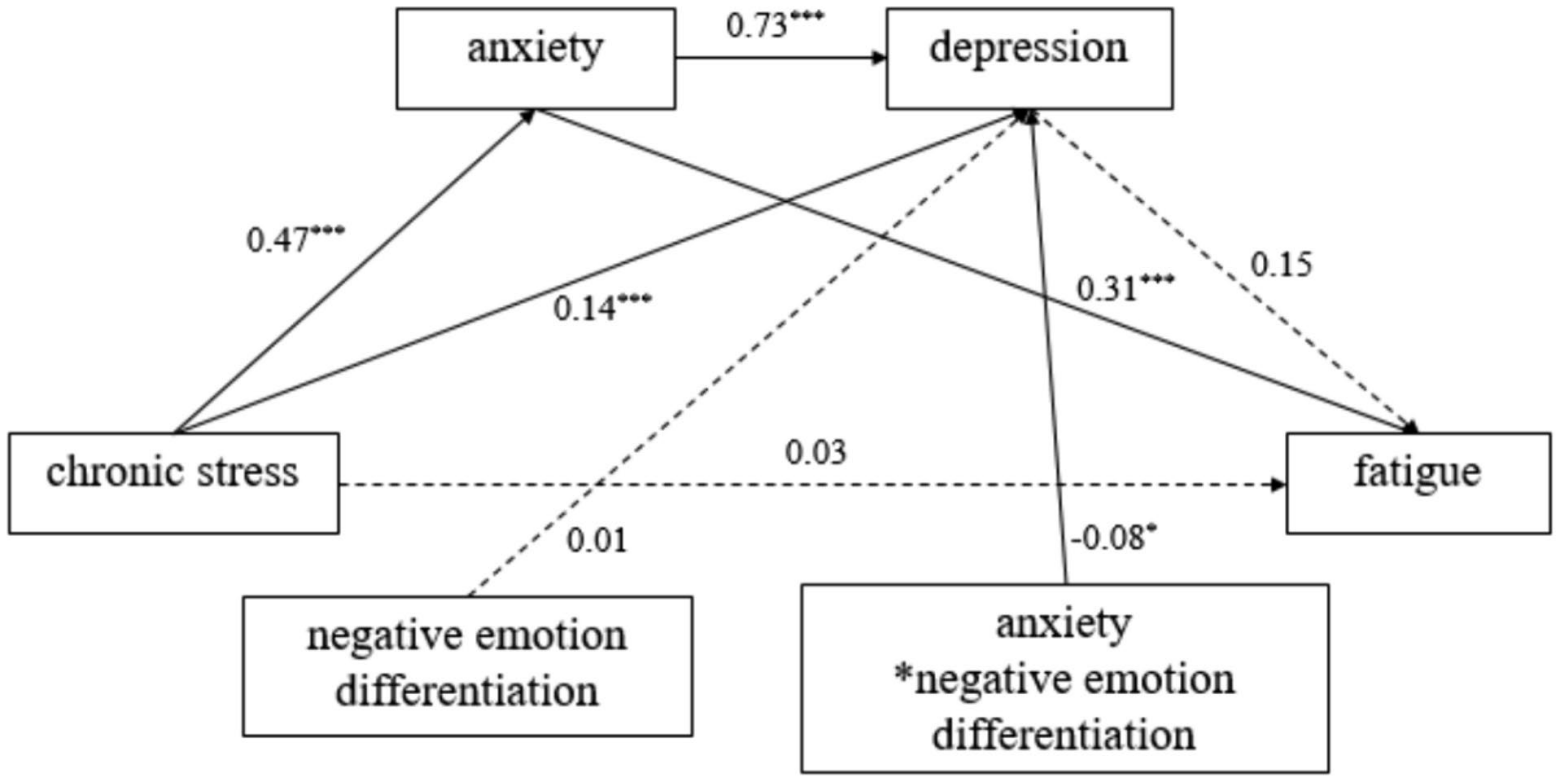
Statistical model.

Simple slope analysis (Table 5) showed that, when negative emotion differentiation was low, anxiety had a significant positive effect on depression (*β* = 0.81, *SE* = 0.04, *95%CIs* = [0.72, 0.89]). When negative emotion differentiation was high, the positive predictive effect of anxiety on depression decreased (*β* = 0.66, *SE* = 0.05, 95% CIs = [0.55, 0.76]). Figure 2 depicts the statistical model of this study.

**Table 5:** Moderating effect of different degrees of negative emotion differentiation.

	*β*	SE	95% confidence interval	
			Lower	Upper
High negative emotion differentiation	0.66	0.05	0.55	0.76
Low negative emotion differentiation	0.81	0.04	0.72	0.89

## 4 Discussion

Prior studies have typically studied the important influence of chronic stress on disease, but have seldom investigated the effect of chronic stress on mental health from the perspective of the underlying psychological mechanisms. This study thus investigated the psychological mechanism by which chronic stress affects fatigue. It yielded three main results. First, this study found that chronic stress, anxiety, depression, and fatigue were significantly positively correlated, indicating that chronic stress, anxiety, depression, and fatigue are closely related. Second, multi-mediation analysis revealed that depression and anxiety played a chain multi-mediating role in the link between chronic stress and fatigue. Finally, negative emotion differentiation was found to play a moderating role in the chained multi-mediation model, indicating that the stronger the negative emotion differentiation, the smaller the influence of anxiety caused by chronic stress on depression, and then the lower the fatigue.

The results of correlation analysis showed that chronic stress was positively correlated with fatigue, depression, and anxiety; this result is consistent with those of prior studies. Decker, Kapila and Wang proposed that chronic stress affects health by changing normal immune function (Decker et al., 2021). According to studies conducted on the workplace, higher chronic workplace stress is likely to lead to a greater risk of psychiatric manifestations, such as burnout and depression (Marin et al., 2011). In addition, according to the reactivity hypothesis, the repeated activation of the stress system has a negative impact on health (Krantz and Manuck, 1984). Therefore, in terms of mental health, the negative effects brought by chronic stress are reflected in negative emotions and the positive correlation between chronic stress and fatigue, depression, and anxiety.

After correlation analysis, this study investigated the mediating role of depression and anxiety between chronic stress and fatigue, and found that anxiety can mediate the influence of chronic stress on fatigue. This result is consistent with the results of prior studies. Changes in stress can change the composition of the ultimate microbiome (Bailey et al., 2011), while changes in the ultimate microbiome have been found to affect individual behavioral responses and anxiety-like behaviors (Cryan and Dinan, 2012), and existing studies have suggested that individuals with high anxiety will feel more fatigue (Polikandrioti et al., 2018). Thus, anxiety mediates the influence of chronic stress on fatigue. In addition, the results of this study show that the chain mediation between depression and anxiety is significant. Anxiety and depression symptoms are often comorbid, in that approximately 85% of depression patients also have obvious anxiety symptoms, while about 90% of anxiety patients have obvious depression (Tiller, 2012). A longitudinal analysis has shown that anxiety and depression are often comorbid, but the relationship between anxiety and personality traits is stronger. Anxiety affects depression over two 3-year intervals, and this influence relationship is nonreciprocal (Wetherell et al., 2001). Nima et al. (2013) discussed the mediating and moderating effects of anxiety, stress, positive emotion, and negative emotion on depression, and found a mediating role for anxiety in the relationship between stress and depression. This is consistent with the results of this study. In the mediating model of the effect of chronic stress on fatigue, anxiety and depression not only play separate mediating roles, but also play chain mediating roles.

According to the present results, the mediating effect of depression between chronic stress and fatigue is critically significant; this result differs from those of prior studies. Chronic stress is a recognized risk factor for depression (Da Estrela et al., 2021). Hammen (2005) reviewed the relationship between stress and depression and explained the role of other factors in the relationship between stress and depression in terms of the diathesis-stress model, which posits that chronic stress has an important impact on depression. Fatigue is considered to be a common residual symptom of major depressive disorder (MDD) (Fava et al., 2014), and there are significant differences between depression patients and healthy people on different fatigue indicators (Pedraz-Petrozzi et al., 2020). This suggests that there is a close relationship between depression and fatigue, and depression may be an important predictor of fatigue. However, this study did not find a mediating role for depression in the link between chronic stress and fatigue. This may be because the influence of depression on fatigue is mainly reflected in the chain mediating role of anxiety and depression. Moreover, fatigue is more easily affected by anxiety than depression, so depression is only critically significant in the mediating role.

According to the diathesis-stress model, emotion differentiation, as an individual psychological quality, can effectively moderate the influence of chronic stress. This study found that negative emotion differentiation plays a moderating role in the effects of chronic stress on fatigue, and constructed a moderated chain mediation model. This is consistent with existing theories and related studies. According to the theory of constructed emotion, emotion is a construction of the world, not the reaction to the world (Barrett, 2006; Barrett, 2016). This theory emphasizes the subjective initiative of individuals, i.e., the ability of individuals to selectively construct emotions, the ability of emotion differentiation. In adolescents, studies have suggested that emotion differentiation is an important influencing factor that moderates stress and psychological problems (Nook et al., 2021). In the military, lower negative emotion differentiation has been found to indicate a stronger association between rumination and depression (Starr et al., 2017). These facts suggest that the effect of emotion differentiation on buffering negative life events may be consistent across groups. Therefore, this study comprehensively investigated this relationship and proved this result. In employees, it was also found that negative emotion differentiation could moderate the influence of chronic stress, indicating that the higher the negative emotion differentiation, the smaller the negative influence of chronic stress, and the less anxiety and depression, and then the less fatigue.

In this study, we investigated the influence of chronic stress on fatigue and its mediating mechanism through depression and anxiety among employees in China. We found that depression and anxiety play important roles and mediate the negative influence of chronic stress. This shows us it is necessary to fully consider individuals’ anxiety and depression symptoms, and carry out prevention and intervention accordingly. Moreover, the chain mediation of anxiety and depression suggests that anxiety plays a more important role than depression, and anxiety caused by chronic stress can not only have a direct impact on fatigue, but also have an indirect impact on fatigue, and thus we should pay more attention to anxiety symptoms. Considering that anxiety symptoms are more stable than depression symptoms and can reflect neuroticism and other personality traits (Wetherell et al., 2001), we should focus first on anxiety symptoms and adopt corresponding effective intervention measures such as mindfulness therapy (Hofmann et al., 2010). At the same time, this study further revealed the important moderating effect of negative emotion differentiation. This indicates that the ability of individuals to accurately distinguish negative emotions is conducive to reducing the negative effects of chronic stress. The improvement of negative emotion differentiation can be achieved through mindfulness and adaptive emotion regulation (Tong and Keng, 2017; Van der Gucht et al., 2019).

Although this study revealed the complex psychological mechanism by which chronic stress affects fatigue, it still has some limitations that should be addressed. First, chronic stress was measured using the PSS, which does not distinguish among different stressors. For employees, work and family are two important stressors, and chronic stress at work may be different from that at home, and thus they could have different effects on fatigue. Therefore, future studies should further consider the different effects of chronic stress under different contexts on fatigue. Second, existing studies have suggested that individuals’ emotion differentiation is constantly fluctuating (Erbas et al., 2018), and negative emotions such as depression and anxiety also change over time. In future studies, time can also be included as a variable. Third, since the scale method may produce interactions between variables due to the different order in which the scales are filled in, it is necessary to verify causality through a series of experiments. Therefore, we intend to conduct empirical experiments on the models involved in the validation of this study in a follow-up study (Kim et al., 2018).

## 5 Conclusion

Through a questionnaire survey of employees in the northwest part of China, this study reached the following conclusions: (1) Chronic stress can significantly predict fatigue. (2) Anxiety plays a significant mediating role in the relationship between chronic stress and fatigue. Depression also plays a critical mediating role in the relationship between chronic stress and fatigue. Anxiety and depression play chain mediating roles between chronic stress and fatigue. (3) Negative emotion differentiation moderates the effect of chronic stress on fatigue by affecting the chain mediation.

## Data availability statement

The original contributions presented in the study are included in the article/supplementary material, further inquiries can be directed to the corresponding author.

## Ethics statement

Ethical review and approval was not required for the study on human participants in accordance with the local legislation and institutional requirements. Written informed consent was provided by the participants.

## Author contributions

WL: Writing – review & editing. HQ: Conceptualization, Methodology, Writing – review & editing, Writing – original draft. HL: Investigation, Methodology, Writing – review & editing, Conceptualization. ZY: Investigation, Writing – review & editing, Methodology. MY: Writing – review & editing, Investigation. CX: Writing – review & editing, Software. XZ: Supervision, Writing – review & editing.
